# Human Papilloma Virus: Current Knowledge and Focus on Oral Health

**DOI:** 10.1155/2021/6631757

**Published:** 2021-02-01

**Authors:** Luca Fiorillo, Gabriele Cervino, Giovanni Surace, Rosa De Stefano, Luigi Laino, Cesare D'Amico, Maria Teresa Fiorillo, Aida Meto, Alan Scott Herford, Alina Vladimirovna Arzukanyan, Gianrico Spagnuolo, Marco Cicciù

**Affiliations:** ^1^Department of Biomedical and Dental Sciences, Morphological and Functional Images, University of Messina, 98100 ME, Italy; ^2^Multidisciplinary Department of Medical-Surgical and Dental Specialties, University of Campania “Luigi Vanvitelli”, 80100 Napoli, Italy; ^3^Clinical Analysis Laboratory “Dott. Francesco Siracusa Rizzi s.r.l.”, Via Nazionale Archi, 89121 RC, Italy; ^4^Unit of Microbiology and Virology, North Health Center ASP 5, Reggio Calabria 89100, Italy; ^5^Department of Conservative, Faculty of Dental Medicine, University of Medicine of Tirana, Tirana, Albania; ^6^Department of Maxillofacial Surgery, Loma Linda University, Loma Linda, CA 92354, USA; ^7^Institute of Dentistry, I.M. Sechenov First Moscow State Medical University, Moscow 119146, Russia; ^8^Department of Neurosciences, Reproductive and Odontostomatological Sciences, University of Naples “Federico II”, 80131 Napoli, Italy

## Abstract

The human papilloma virus (HPV) is responsible for different pathological manifestations in humans. This agent gives rise to lesions of different types and in different areas of the organism, including the oral cavity. The aim of this study is to show which are the main diseases for which HPV is responsible and to bring to light some of the interceptive and therapeutic strategies. The analysis was conducted by consulting the major scientific databases with the aim of obtaining information on the characteristics of oral HPV and its management; furthermore, the literature was supported by some clinical cases proposed by the authors. The role of dentistry is essential in the early diagnosis of this type of pathologies and above all in knowing how to direct patients towards a path that can lead to patient management, especially in the event that these lesions have a malignant potential. Enhancing the knowledge and role of dentistry can lead to early diagnosis of this type of injury, intercepting a pathology that could have multiorgan implications.

## 1. Introduction

Papilloma viruses (from the Latin “papilla” meaning pustule and from the Greek suffix “oma”) are classified in the Papillomaviridae family, officially recognized by the International Committee on Taxonomy of Viruses (ICTV). The human papilloma virus (HPV) is a small-sized DNA virus (diameter 50-55 nm) without envelope, which is resistant to heat, acids, and ether. Currently, over 120 types of HPV have been identified, identifiable in 16 different genera based on biological properties and the organization of the genome [1–3].

Papilloma viruses have a high tropism for stratified squamous epithelial cells and replicate only in differentiating epithelial cells of the skin and mucous [2].

The infection begins with the entry, following small wounds or superficial abrasions of the host's mucosa, of a viral particle into a cell of the basal epithelial layer called keratinocyte characterized by a marked proliferative activity. Once the virus has penetrated into the keratinocyte, it could remain in a latent state or undergo active replication. This virus is responsible for causing lesions of the affected tissues [4–6].

Cancer of the uterine cervix, whose incidence is about 600,000 new cases every year with rates of 40 cases per 100,000 women in some geographic areas at higher risk such as Central America, South America, and Africa, is the first tumor recognized by the World Health Organization with infectious etiology. The etiological agent is the human papillomavirus responsible for most sexually transmitted diseases. It is estimated that about 75% of women will contract an HPV infection in their lifetime, but only a small percentage of these infections will become persistent and may lead to cell atypia up to the development of carcinoma. The role of the virus in oral carcinogenesis is highly controversial, but it is now believed that HPV infection, in particular HPV 16, is important in the pathogenesis of oropharyngeal cancer; oral carcinoma accounts for more than 90% of all malignant neoplasms of the mouth. It most frequently affects individuals over fifty years of age and males to a greater extent than females in a ratio of about 2 : 1 [7–10].

The HPV involved in skin infections being resistant to heat and drying can be transmitted by means of fomites following prolonged contact with contaminated clothing, unlike the types that infect the genital tract for which sexual transmission prevails as demonstrated by failure to detect the virus in individuals who have not had sexual intercourse. Perinatal transmission in children born to mothers with genital warts is very rare. HPV is a virus that is frequently found in the genitals, and it is through oral intercourse that it passes from the genitals to the mouth. In rare cases, the virus that is localized in some cells of the oral mucosa changes them and transforms them into malignant tumors. However, the high association of patients with oral carcinoma with papilloma virus infection was observed. For this reason, early diagnosis of oral infections becomes essential [3].

The purpose of this study is to clarify the main features of HPV and its signs, symptoms, and management in dentistry.

## 2. Materials and Methods

**Figure 1:**
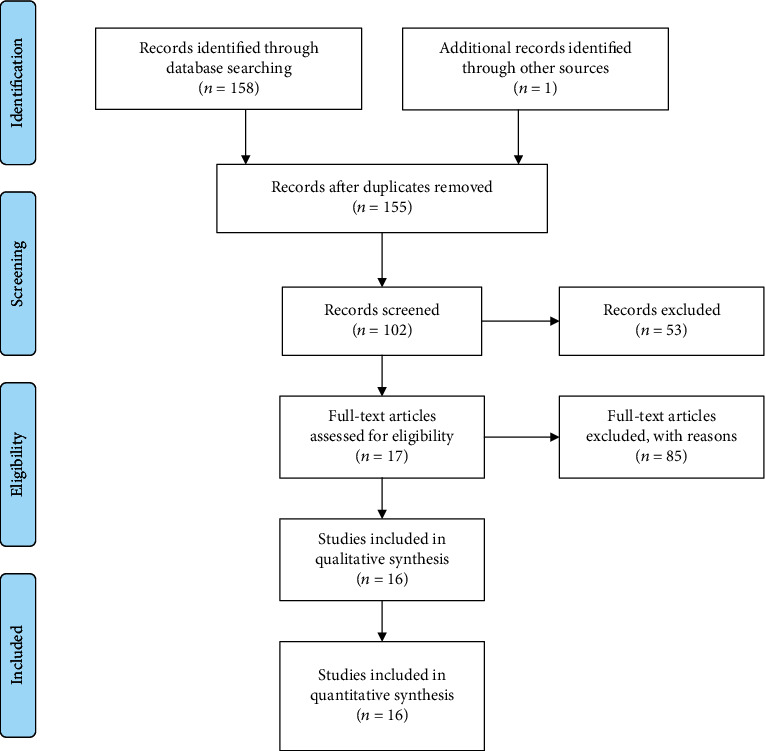
Identification, screening, and eligibility phase of literature, according to PRISMA.

Data collection for this scientific article was conducted using different search engines: PubMed, Scopus, and Web of Science. The aim of the authors was to bring to light what were the main characteristics of this virus and above all the potential risk to the health of patients. The search was carried out in the different scientific databases and with the help of textbooks. This involved screening and analyzing the data independently by the authors. Any doubts and clarifications were subsequently critically evaluated by some expert reviewers (M.C. and L.L.) (Figure 1) [11–26].

The narrative review of the literature was also supported by some clinical cases managed by the authors with the aim of giving better visibility to the scientific manuscript, with a particular case also of negative PCR and histological positivity. The analysis of the literature provided a large number of results that were screened and read independently by the authors. The individual manuscripts were evaluated on the basis of the title and abstract, to evaluate a first inclusion (removal of articles that were repeated or not available in English or as full text), and subsequently, they were subjected to reading of the full-text. In this way, it was possible to analyze in detail the individual results and include only those that contained sufficient data to conduct a narrative review.

## 3. Results

### 3.1. HPV Classification

The IARC (International Agency for Research on Cancer) in its latest epidemiological classification divides HPV based on their oncogenicity in mankind into the following:

- Group 1: carcinogenic to humans (HPV 16, 18, 31, 33, 35, 39, 45, 51, 56, 58, and 59)
- Group 2A: probably carcinogenic to humans (HPV 68)
- Group 2B: possibly carcinogenic to humans (HPV 26, 53, 66, 67, 70, 73, and 82)
- Group 3: not classifiable as to its carcinogenicity to humans (HPV 6 and 11) (Table 1).

Based on their tropism, papillomaviruses are divided into two broad categories:

Cutaneous HPV is responsible for skin lesions such as common or vulgar warts (these are the most common forms) which are frequently localized in the hands and feet and which manifest themselves in the form of white-greyish or brown flat or raised papules. The genotypes most frequently associated with warts are types 1, 2, 3, 4, and 7. Cutaneous HPV genotypes 5, 8, 9, 12, 14, 15, 17, 19, 20, 47, and 49 are associated with epidermodysplasia Lewandowsky-Lutz verruciform. This is a rare familiar dermatosis, with chronic evolution, characterized by a dissemination of numerous papular and macular elements, which usually appears in childhood and which within 20-30 years of onset can encounter a neoplastic transformation (first bowenoid carcinoma in situ and subsequently squamous cell epithelioma) which manifests itself in the form of flat warts and reddish macules and which can also degenerate into squamous cell carcinomas [27, 28].

Mucosal HPV is responsible for benign mucous lesions that mainly include sharp and flat warts that arise in the female and male genitalia, the urethra, the perianal area, and the anus. The genotypes associated with these infections are genotype 6 and genotype 11.

Malignant mucosal lesions were associated with high-risk genotypes and characterized by low-grade (LSIL) or high-grade (HSIL) squamous intraepithelial lesions (SIL) which on the Pap test show characteristic cells with nuclear atypia and perinuclear halo called koilocytes [29–32].

### 3.2. Diagnostic Methods

The tests available for the laboratory diagnosis of HPV infections, given the lack of effective culture methods, are numerous and differ in applicability on biological material, sensitivity, and specificity. They can be divided into cytological, immunohistochemical, and molecular methods.

Cytology uses the Papanicolaou stain of smears of samples taken from the uterine cervix (Pap test). This technique consists of evaluating any alterations present in HPV-infected cells. Squamous cell abnormalities are divided into four categories:

1. ASC (atypical squamous cells) with the subcategory ASC-US (atypical squamous cells of undetermined significance) which includes cellular alterations suggestive of SIL (intraepithelial squamous lesion) and the ASC-subcategory H (atypical squamous cells) which does not exclude H-SIL (high-grade squamous intraepithelial lesion)
2. LGSIL (low-grade squamous intraepithelial lesion) and low-grade intraepithelial squamous cell lesions
3. HGSIL (high-grade squamous intraepithelial lesion) and high-grade squamous cell lesions
4. Squamous cell carcinoma [29, 33, 34]

Immunohistochemical methods use fresh or fixed cytological material that is incubated with polyclonal or monoclonal antibodies, directed against major and minor capsid antigens, capable of recognizing different subtypes. Subsequently, biotinylated antibodies linked to the avidin-peroxidase complex and capable of binding to the primary antibody are used. The final phase of the revelation involves the addition of specific chromogens and countercoloring with hematoxylin. The nuclei of infected cells show a red or brown color depending on the chromogen used. The limits of this method are related to low sensitivity, especially in cases where the capsid antigens are not expressed [35, 36].

Molecular methods allow the identification of viral DNA with relative genotyping in preparations obtained from different biological samples. In women, superficial cells of the cervical mucosa, fresh cervical biopsies or those fixed in formalin or paraffin, and vulvar papillary biopsies are used as biological samples. In humans, the superficial cells of the glans, urine, and seminal fluid are used [37].

Except for the paraffinized samples which must be treated with xylene and washed with ethanol, all the others are first treated with proteinase K at different concentrations based on the type and quantity of cellular material to be digested and subsequently lysed with isopropanol. For the extraction of viral DNA to chemical methods, which use phenol and chloroform, those on the column are preferred today, which involve a lysis step, a binding of the nucleic acid to the column membrane, repeated washing, and a final elution of the acid. Nucleic acid is purified with a vacuum system. Once the purified DNA has been obtained, the three main methods used are direct hybridization, signal amplification, and gene amplification (PCR). Direct hybridization uses appropriately labeled probes that bind specifically to homologous DNA sequences contained in the sample to be analyzed. The probe-DNA binding of the sample is then revealed either by using radioactive tracers or by using streptavidin labeled with alkaline phosphatase which is made to act on a suitable substrate [38–40]. The method of amplification of the nonradioactive signal mostly used and approved by the FDA (U.S. Food and Drug Administration) is “Hybrid Capture” (Digene, Beltsville, MD). It uses specific RNA probes that by binding to viral DNA, if present, form RNA: DNA hybrids that are recognized by universal antibodies linked to the solid phase (microplate). The detection takes place in chemiluminescence with an identification threshold equal to one picogram of viral DNA per ml. The method only allows us to distinguish between high-risk and low-risk genotype and does not identify the single specific genotype. Furthermore, it is not possible to identify coinfections, and the determination of the viral load is semiquantitative (through a chemiluminescence intensity gradient). Since an HPV infection results in both humoral and cell-mediated immune responses, the serological evaluation of anti-HPV antibodies can be used as a virus exposure test. However, the sensitivity is low (50-60%) while the specificity is about 90%. The tests described in the literature are of the immunoenzymatic or radioimmunoassay type based on the use of type-specific VLPs adsorbed on microplates [41].

Gene amplification (polymerase chain reaction) is a PCR technology invented in April 1983 by Kary B. Mullis who received the Nobel Prize in Chemistry for this in 1993. It is a biomolecular technique that allows the exponential amplification in vitro of a given DNA sequence defined as target DNA [42, 43].

#### 3.2.1. PCR

The essential components of the PCR reaction mixture are a thermostable DNA polymerase, the “primers” which are single-stranded DNA oligonucleotides complementary to the two ends of the target segment, the deoxyribonucleotide triphosphates (dNTPs), the magnesium ions, and the target DNA. The amplification reaction has three phases:

1. Denaturation (separation): the target dsDNA (double-stranded DNA) is denatured at about 95°C and is converted into single-stranded DNA
2. Pairing (annealing): the complementary oligonucleotide “primers” at the two ends 3′ of the sequence to be amplified hybridize with the two denatured filaments; their sequence is oriented so as to be able to guide DNA polymerization in the stretch between the two regions to which they associate
3. Extension: the oligonucleotide “primers”, in the presence of the four triphosphate deoxynucleotides and a DNA polymerase, are each extended towards the other but on two different complementary chains leading to the synthesis of two molecules of ds-DNA copies of the delimited target region from the primers. The cycle can also be repeated up to 60 times leading to the exponential amplification of the “target” DNA (target DNA) [44]

Different types of primers designed to amplify highly conserved sequences of the L1, E1, or E6/E7 region of the viral genome can be used for DNA detection of papillomavirus. The most commonly used are the consensus primers directed towards the L1 region of the virus (GP5+/GP6+, MY09/MY11). MY09/MY11 identifies 25 HPV genotypes and generate a 450 bp fragment, while GP5+/GP6+ generates a 140 bp fragment identifying an HPV spectrum similar but not identical to MY09/MY11. The primers directed towards the E6/E7 region that identify only the high-risk genotypes are the pU-IM/pU2R which generate a fragment of 233-268 bp on the E6 region. Once the amplification reaction is completed, the type-specific identification is carried out which can be carried out using various methods:

- RFLP (restriction fragment length polymorphism) which uses restriction enzymes that generate genotype-specific bands. Interpretation is somewhat difficult in the case of packaging
- ELISA (enzyme-linked immunoabsorbent assay) which uses type-specific oligonucleotide probes in microplates and a colorimetric reaction to highlight the hybridization
- LIPA (line probe assay) which identifies amplified DNA by selective hybridization with membrane-immobilized oligonucleotides. The method is sensitive and also has a good level of discrimination in the packaging
- Sequencing that uses terminator nucleotides in which the DNA sequence is determined by enzymatic synthesis of polynucleotide chains interrupted with the addition of dideoxy terminator nucleotides. It allows the identification of the viral genotype by comparing sequences using an automatic sequencer [35]

The viral load is determined using real-time amplification techniques based on the use of highly specific probes which emit a fluorescent signal when probe/DNA hybridization occurs and which allow an accurate quantitative analysis to be performed, also detecting a low number of DNA copies. The fluorescent signal is read in real time at each amplification cycle, generating curves which, compared to a standard, allow for a quantitative evaluation of the viral load. Several studies have shown a statistically valid correlation between viral load and risk of developing cervical cancer and high sensitivity, reproducibility, and speed of execution [45].

The determination of the mRNA transcripts of E6/E7 oncoproteins, a useful prognostic indicator of the progression of lesions towards cervicocarcinoma, uses NASBA (nucleic acid sequence-based amplification) technology based on isothermal amplification (conducted at 41°C) of the target RNA and on the use of particular probes defined as molecular beacons capable of emitting fluorescence at two different wavelengths when a complementary sequence is present [35, 45–47].

### 3.3. Transmission Route

Transmission of the infection occurs with three different methods of contact:

- Direct horizontal contact (saliva-saliva or genital mucous membranes during sexual intercourse): oral sexual contact is certainly one of the most frequent ways of transmission of the papilloma virus; a relationship has been demonstrated between the presence of HPV in the oral cavity and the age of onset of sexual activity in the young; the presence of oral HPV is also more evident in couples in which oral sex is practiced, compared to couples in which only genital sex is practiced; a 10 times higher risk of oral HPV infection was then calculated in patients who have had more than 20 sexual partners in their lifetime compared to individuals with fewer than 3 partners [48]
- Indirect contact (through contaminated medical instruments, utensils, or linen)
- Vertical maternal-fetal transmission (during childbirth or postnatal) [49, 50]

### 3.4. Oral Signs and Symptoms

**Figure 2:**
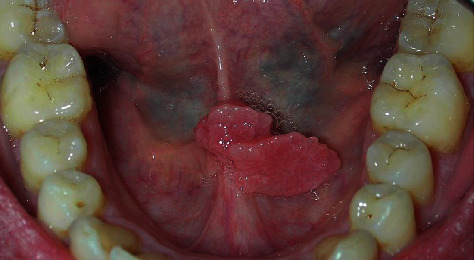
Oral HPV lesion. With gentle permission from Prof. L. Laino.

**Figure 3:**
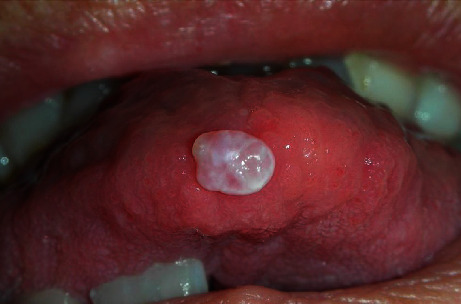
Oral HPV lesion. With gentle permission from Prof. L. Laino.

- Subclinical infection: the presence of HPV in a clinically normal mucosa is found in about 10% of subjects, which represent a potential reservoir for the transmission of infection
- Squamous papilloma: it is associated with HPV 6 and 11, and it is the most common manifestation of oral HPV infection; it is localized on the nonkeratinized mucosa (lingual belly, soft palate) or keratinized (hard palate) and appears as an exophytic neoformation with a cauliflower surface. From the histological point of view, it is composed of a connective axis with a villous and papillary morphology (arborescent appearance) covered by a paving epithelium which presents a notable hyperkeratosis and acanthosis (verrucous hyperplasia); at the cellular level, there is a clear vacuolisation of the cells of the spinous layer (koilocytosis) characteristic of HPV infections
- Verruca vulgaris: it is associated with HPV 2 and 4, is rare in the oral mucosa, and is localized at the level of the keratinized mucosa (lingual dorsum, adherent gingiva); it is found more frequently in children, as a consequence of self-inoculation, and is generally self-limited, resolving in less than 2 years
- Condyloma acuminata: it is associated with HPV 2, 6, and 11, and it is extremely contagious and is often found associated with genital warts. It is localized on the nonkeratinized mucosa (lingual belly, soft palate) and has a wider implant base than papillomas and warts and a more pinkish color as it is often devoid of hyperkeratosis
- Squamous cell carcinoma: the role of the virus in carcinogenesis of the oral cavity is highly controversial, but it is now believed that HPV infection, in particular HPV 16, is important in the pathogenesis of oropharyngeal cancer (cancer of the back of the mouth); in fact, oropharyngeal tumors are 70% HPV positive (HPV 16), and compared to the healthy mucosa, HPV infection in these tumors is almost 13 times more frequent [51–53]. There is also a whole literature that reports the existence of a significant relationship between the incidence of oropharyngeal cancer and “sexual behavior” (habit of oral sex and the presence of multiple partners). Today, it is hypothesized that the tumor associated with HPV infection follows a different pattern compared to the “classic” tumor of the oral cavity which is generally linked to known risk factors (smoking and alcohol) (Figures 2 and 3) [54–57]

**Figure 4:**
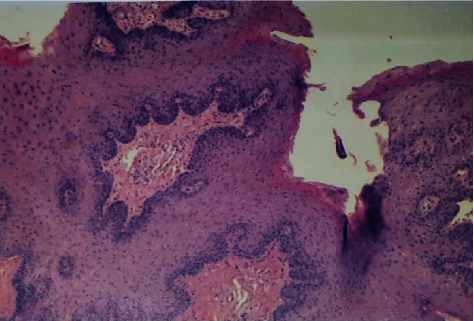
Histology of the lesions. Diagnosis of squamous cell papilloma (20 × 20 magnification).

**Figure 5:**
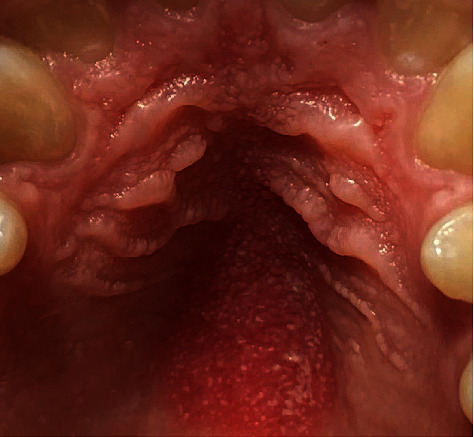
Clinical photograph of the palatal lesion. With gentle permission from Dr. Luca Fiorillo.

In Figures 4 and 5, a singular case can be observed (a 26-year-old female, nonsmoker). The patient comes to the clinician's observation complaining of “a strange coloration of the palate.” The area had never shown symptoms and was probably present for a couple of months. Physical examination revealed a nonhomogeneous/granular erythematous area, with a slightly raised but not vegetating appearance. On palpation, it appeared of regular consistency, not bleeding and not painful. A double biopsy exam is then planned. After disinfection with chlorhexidine 0.20%, locoregional anesthesia is performed and a double lonsanga of the partial thickness mucosa is incised. Haemostasis maneuvers and 1 week control. The biopsy tissues are arranged in two different containers: (i) 10% formalin (for histology) and (ii) temperature-controlled physiological solution (for PCR). Biopsy reported “palatal neoformation,” 0.6 cm × 0.2 sample, and squamous cell papilloma.

### 3.5. Therapy

Contrary to other viral infectious conditions that respond to drug therapy, for HPV infection, there are no active ingredients available that can eradicate the infection or induce regression of clinical lesions where present. Some drugs have been tested for topical and/or systemic use for the treatment of genital lesions, but to date there are no available treatment for genital lesions. For oral subclinical infection, similar to genital infection, no treatment is provided; none of the antiviral drugs tested (for example, acyclovir and ribavirin) proved effective in eliminating the infection. The management provides for the patient's follow-up and the repetition of the HPV test 8-12 months after the first detection and periodically to verify the eradication of the infection by the immune system. In the presence of HPV-related lesions, their treatment is mainly surgical as they are not responsive to topical application or systemic administration of cytotoxic or immunomodulating drugs. The recommended surgical treatment is the excisional one using a cold blade scalpel, quantum resonance scalpel, or laser, which allows performing the histological examination of the piece. The application of low-level laser therapy (LLLT) protocols to the oral manifestations of viral infections and in particular to the relapses of cold sores (herpes simplex virus 1) [58] is justified by the accelerated healing of the wounds with simultaneous reduction of pain, both key factors in the recurrence of the labial resulting - not often painful and subject to slow healing processes. These aspects are associated with the stimulating action of the patient's immune response, reported for the LLLT [59–61]. It is good to keep in mind that, by virtue of the multifocality of the HPV oropharyngeal infection, the surgical removal of the lesion does not guarantee the eradication of the infection, since HPV-DNA could persist in the adjacent healthy mucosa [62, 63].

The new therapeutic perspectives concern the possibility of using “therapeutic vaccines,” i.e., real molecules capable of arresting tumor growth linked to vectors directed against some viral antigens and therefore directed against positive HPV CSCs [64–67].

There are currently three prophylactic vaccines already evaluated by randomized clinical trials, but only the last two have been released:

- Monovalent anti-HPV 16 vaccine
- Direct quadrivalent vaccine against HPV 6, 11, 16, and 18
- Bivalent anti-HPV vaccine 16 and 18 [68, 69]

The HPV vaccine also protects against oral infection caused by the virus, which causes more than 30% of oropharyngeal cancers. So much so as to lower the prevalence of infection by 88% in those who have carried out at least one dose of vaccine compared to those who have decided not to get vaccinated, the HPV vaccine is indicated for the prevention of cancer 5of the cervix, vulva, vagina, anus, and penis because it has been shown to be able to prevent infection of the most aggressive HPV strains, those that cause warts or cancer.

## 4. Discussion

The most common malignant neoplasm of the oral cavity is squamous cell carcinoma. According to recent estimates, both the annual incidence rates of the disease and the mortality rates have not decreased in recent years, still representing a serious public health problem. The disease is in fact characterized by considerable local aggression and an often-poor prognosis, with a five-year survival ranging from 59.4% to 67%. The initial hypotheses about a possible etiological role of the virus in oral and oropharyngeal carcinogenesis, dating back to the early 1980s, were subsequently supported by the following evidence:

- The epithelial tropism of the virus
- The confirmed etiological role of HPV-HR in almost all squamous cell carcinomas of the uterine cervix and in about 40% of cases of other anogenital squamous cell carcinomas
- The histological similarities between the lining epithelia of the two mucous districts
- Despite the histological similarities of the two epithelia, it should however be reiterated that the environment of the uterine cervix is much more receptive to the permanence and entry of the virus than any other anatomical site
- The role of the virus in oral carcinogenesis is highly controversial [14]

HPV-associated cancer, on the other hand, occurs in young subjects, mainly men, is independent of traditional risk factors, and is instead correlated with sexual behavior (number of partners, age of sexual onset, and orgenital sexual practices), mainly involving oropharynx (e.g., palatine pillars, tonsils, lingual base, lower face of the soft palate, pendulous veil, and posterior pharynx wall) (Table 2) [70].

In some cases, a combined protocol may be required for diagnosis. The biopsy intervention can be split and stored in a different way so as to perform a double analysis with a single surgical intervention on the patient. Keeping part of the excised tissue in formalin and part in physiological can allow performing both a histological examination (formalin) and a PCR test to identify traces of DNA of certain viral strains. However, there are reported cases where PCR is negative and histology is positive (Figures 4 and 5) [70]. This may be related to the fact that a limited number of viral strains are considered in the PCR test [71].

The virus is normally found in the genital and perianal area. If these come into contact with the mouth, the passage into the oropharyngeal mucosa is quite obvious. In most cases, the infection resolves spontaneously and the virus disappears. In other cases, however, it lurks and can give rise to pretumor lesions. The cigarette itself is a risk factor for various forms of airway cancer, including the oropharynx. Smoking aggravates the state of inflammation and could facilitate the evolution of tissues towards cancer. It is possible that smoking also acts on the defensive capacities of the immune system at the level of the mucous membranes: marijuana smoke, for example, has been associated with a greater risk of oral HPV infection, and it is believed precisely because of its immunodepressive activity. It should be checked whether the same also applies to the cigarette one. Limiting the number of oral sex partners, not smoking, and getting vaccinated against HPV are three strategies for reducing risk [50, 72].

## 5. Conclusions

Patients at risk or those already affected by the disease must undergo periodic checks by both ENT (ear, nose, and throat)/dentist (ENT/D) and gynecologist. The ENT/D and the gynecologist, in synergy, could schedule follow-ups for these patients at risk, even for couples. The sensation of an oral-pharyngeal foreign body, the pain or burning of the oral mucosa, and the bleeding (even if rare) immediately require a specialist visit and therefore the appropriate care of the case. In recent decades, the amount of information regarding HPV infection and its oncogenic potential has grown considerably, hand in hand with the progress of molecular biology techniques applied to the diagnostic, preventive, and therapeutic management of HPV-induced lesions. Understanding the interactions between the viral agent and the host has provided new targets for the control of the infection and laid the foundation for the formulation of new targeted therapeutic protocols. It is essential to emphasize that the general population has little knowledge about the methods of transmission and clinical presentation of HPV infection, an aspect mostly due to the scarcity of information campaigns, the implementation of which is desirable in order to achieve better infection control. While waiting for these information and prevention programs to be implemented, the role of the dentist is of great importance, for head and neck cancer detection too.

## Figures and Tables

**Table 1:** HPV subdivision on its cancerogenicity and pathological signs.

Type of lesion	HPV genotype
*Cutaneous lesions*	
Vulgar wart	1, 2, 3, 4, 7, 10, 27, 28, 29, 41
Skin carcinomas	5, 8, 14, 17, 20, 47
*Mucosal lesions*	
Respiratory papilloma	6, 11
Conjunctive papilloma	6, 11
*Oral mucosa lesions*	
Labial lesions	2
Oropharyngeal carcinoma	16, 33
Focal epithelial hyperplasia	13, 32
*Anogenital mucous lesions*	
Sharp and flat warts	6, 11, 42, 43, 44, 54, 55
Dysplastic lesions: Low grade High grade	16, 18, 45, 56, 58, 31, 33, 35, 51, 52
Giant condyloma	6, 11, 16
Carcinomas of the cervix	16, 18, 31, 33, 35, 39, 45, 51, 52, 56, 58, 59, 66, 68
Anal cancer vulvar cancer	16
Bowenoid papulosis	16

**Table 2:** HPV oral signs.

Definition	Clinical signs	Description	% of HPV infections
Subclinical	Normal mucosae	No clinical or histopathological lesions HPV-DNA revealed by the molecular biology technique	0-81%
Clinical	Benign lesion	Squamous cell papilloma	5-62%
Associated	Potentially malignant lesion	Oral leukoplakia Oral erythroplakia Oral lichen planus Leukoplakia proliferative wart	22-100% 0-54% 11-60% 0-89%
	Malignant lesion	Squamous cell carcinoma Verrucous carcinoma	0-38% 0-7.7%

## Data Availability

Data are free and available upon request to corresponding authors.
